# Displacement-Sensing Method Based on Residual Scaling for One-Shot MMF Specklegram Prediction

**DOI:** 10.3390/s25051434

**Published:** 2025-02-26

**Authors:** Bohao Shen, Jianzhi Li

**Affiliations:** 1 School of Mechanical Engineering, Shijiazhuang Tiedao University, Shijiazhuang 050043, China; shenbohao0105@163.com; 2 Key Laboratory of Structural Health Monitoring and Control, Shijiazhuang Tiedao University, Shijiazhuang 050043, China

**Keywords:** multimode fiber specklegram, displacement sensor, high resolution and wide range, deep learning, residual scaling, one-shot prediction, temperature immunity

## Abstract

A high-resolution and wide measurement range displacement sensing method based on multimode fiber (MMF) is proposed. To achieve a high-resolution displacement detection model, a one-shot dataset was constructed by collecting MMF specklegram images for 1801 displacements with resolution of 0.01 mm. This work modifies the fully connected layer of a residual network (ResNet) to achieve displacement prediction and applies residual scaling to reduce prediction errors in the one-shot learning task. Under stable environmental conditions, experimental results show that this method achieves an average error as low as 0.0083 mm in displacement prediction with resolution of 0.01 mm; meanwhile, the measurement range reaches 18 mm. Additionally, the model trained on a 0.01 mm resolution dataset was evaluated on a specklegram dataset with a resolution of 0.005 mm for its generalization ability, yielding an average error of 0.0138 mm. Regression evaluation metrics demonstrate that the proposed model has a significant improvement over other displacement-sensing methods based on MMF specklegrams, with prediction errors approximately three times lower than ResNet. Additionally, temperature immunity was studied within an 18 mm measurement range under a temperature range from 21.25 °C to 22.35 °C; the MMF displacement sensor demonstrates a dispersion of 5.08%, an average nonlinearity of 7.71% and a hysteresis of 6.13%. These findings demonstrate the potential of this method for high-performance displacement-sensing in practical applications.

## 1. Introduction

Displacement sensing is critical in various fields, including structural health monitoring, robotics, and precision manufacturing. MMF has emerged as a promising sensor due to its ability to generate complex and highly sensitive specklegram patterns in response to external perturbations. Notably, MMF can achieve high-sensitivity displacement responses, making it particularly suitable for precise measurements. However, the challenge lies in accurately recognizing and interpreting these complex specklegrams under high-resolution displacement conditions. Advanced MMF specklegram-recognition techniques are essential for a high-resolution displacement-sensing application of MMF. Recent advances in specklegram analysis, especially deep learning-based demodulation, have demonstrated significant potential for improving both resolution and robustness [1].

To apply vertical displacement to the MMF, a piezoelectric actuator was used to achieve sub-micron displacement variations in the optical fiber [2,3,4,5]. Yu et al. [2] initially proposed employing the Normalized Intensity Inner Product Correlation (NIPC) algorithm to establish the relationship between displacement variations and specklegram variations, leveraging the algorithm’s ability to compute similarity between two specklegram images. They detected a minimum displacement variation of 0.1 μm, with a maximum displacement range of 20 μm. Rohollahnejad et al. [3] achieved a minimum displacement resolution of 0.02 mm using the NIPC. Fujiwara et al. [4] employed image segmentation to locally quantify variations in the specklegram image, achieving a displacement resolution of 0.01 mm over a measurement range of 0.35 mm. To exploit specklegram features for displacement detection, Lomer et al. [5] applied the Grey Level Co-occurrence Matrix (GLCM) algorithm, achieving a displacement resolution of 0.1 μm within a 20 μm measurement range, and 0.03 μm within a 0.5 μm measurement range. This algorithm reflects displacement variations in the optical fiber by analyzing the texture characteristics of the specklegram image, offering lower computational complexity and better detection accuracy compared to the NIPC algorithm. In addition to detecting vertical displacements using a piezoelectric actuator, Liu et al. [6] employed a high-precision translation stage to apply relative lateral displacements between single-mode fiber and multimode fiber, achieving vertical displacement detection with resolution of 2 μm within a 50 μm measurement range using the GLCM algorithm. The traditional methods reach a maximum of 0.35 mm. In summary, within both the NIPC and GLCM methods, their measurement ranges are narrowed with a sub-micron vertical displacement detection.

In deep learning methodologies, convolutional neural networks (CNNs) demonstrate robust feature extraction capabilities. By extracting features from specklegram images, CNNs achieve high recognition accuracy in specklegram-recognition tasks. Liu et al. [7] employed a VGG [8] network architecture to achieve a resolution of 0.5 mm within a horizontal displacement range of 10 mm. Sun et al. [9] utilized a ResNet [10] to achieve displacement recognition with resolution of 0.8 mm within an 8 mm range in two directions. Li et al. [11] also employed the VGG network and successfully implemented regression-based displacement prediction, achieving resolution of 0.1 mm within a 2 mm measurement range. Subsequently, they proposed a method using one-dimensional convolutional neural networks to extract texture features, which further reduced recognition errors [12]. Wei et al. [13] achieved displacement detection of ten steps using a CNN with two convolutional blocks and two fully connected layers. Although CNNs have shown significant progress in improving the accuracy of MMF specklegram recognition, the range and resolution of displacement measurement are mutually constrained, making it difficult to achieve both a large measurement range and high resolution simultaneously. In addition, several other MMF optic sensors also utilize CNN methods, such as multi-point localization sensors [14], tip deflection sensors [15], bending sensors [16], planar tactile sensors [17], and torsion sensors [18]. Most of these CNN-based approaches employ classifiers [7,9,13,14,15,16,17], while others, such as [11,12,18], utilize regression models to achieve predictive capabilities. These methods have demonstrated high recognition accuracy, although some fail to achieve a high resolution within their measured range.

Meanwhile, for the present work, multiple specklegram images were captured for each physical quantity, or multiple cycles of data collection were performed within a single dataset. As a result, each category (or attribute) in the dataset contains numerous samples. While this approach mitigates overfitting and improves model accuracy, it also makes the data collection process more cumbersome, and results in a limited recognition range and resolution of the MMF sensor. To simplify the data collection process, only one sample is collected for each movement of the translation stage, without repeated sampling. The dataset constructed in this manner ensures that each specklegram pattern is unique; it is called a one-shot dataset, or one-shot learning [19,20]. This one-shot dataset significantly enhances the resolution and density of the collected data within the same level of work efficiency. However, achieving a high-precision one-shot data prediction is a challenging task due to certain issues, such as poor learning stability or overfitting in the one-shot MMF specklegrams dataset which results in prediction errors.

In this work, these problems of existing displacement-sensing techniques are addressed by introducing several strategies used to enhance its prediction accuracy, measurement range, and resolution. This approach constructs a one-shot dataset (1801 displacement values, with resolution of 0.01 mm) to predict the displacement by training a deep learning model. To enhance the accuracy of ResNet, this work applied residual scalar in each residual connection. Experimental results show a notable reduction in the average prediction error. The study demonstrates that the proposed method achieves high-precision displacement prediction even under one-shot data conditions. Finally, to enable displacement sensing under varying temperature conditions, multiple groups of specklegrams were acquired through one-shot sampling across the different temperatures. Subsequently, specklegrams within the same temperature range were predicted, yielding reliable displacement-sensing outcomes.

## 2. Methods

### 2.1. Experimental Setup and Data Processing

When coherent light is coupled into an MMF, an output specklegram emerges at the end face of the fiber. This phenomenon arises from the interference of modes propagating through the core, with the number of speckles being directly related to the number of transmitted modes [21]. Variations in the relative phases and energy distribution among these modes lead to fluctuations in both the intensity and spatial distribution of the speckle patterns over time [22].

According to [2], the output specklegram projected onto an xy-plane can be described by the detected light intensity I(x,y), represented as

(1) $$I\left(x,y\right)=\sum_{m=0}^{M}\sum_{n=0}^{M}a_{m}a_{n}e^{j(\phi_{m}-\phi_{n})}$$

where M is the total number of propagating modes, and a and ϕ denote the amplitude and phase of the *m*-th or *n*-th mode, respectively.

Conversely, specklegrams exhibit properties which are valuable for fiber sensing applications. External stimuli, such as mechanical vibrations, bending, or changes in temperature, can modulate the characteristics of the propagating modes, gradually altering the specklegram. In this context, the output specklegram becomes indicative of the fiber’s environmental condition [23]. By analyzing the intensity variation of specklegrams, the displacement at a specific point on the fiber can be determined. A neural network model, trained on a large dataset of specklegram images corresponding to various displacements, can then predict fiber displacement, enabling displacement recognition based on MMF specklegrams.

To acquire the datasets, an experimental system was established with a laser source, MMF, and a high-resolution CCD camera to capture specklegrams. The MMF was subjected to controlled displacements via a precision translation stage, and the corresponding specklegrams were recorded. The experimental setup details are as follows:

(1) Light Source: A 530 nm laser was coupled into the MMF through an SMA905 (Optics Experiment and Basie Teaching Technology Co., Ltd., Guangzhou, China) interface. The laser power was adjustable, with 25 mW used for clear specklegram images.

(2) Multimode Fiber: A one-meter step-index MMF (Weiguangxing Technology Co., Ltd., Shenzhen, China) with a 50 µm core diameter and 0.22 numerical aperture (NA) was used. A fiber-optic collimator (FOC) (Fibestar Technology Co., Ltd., Beijing, China) was utilized at the output end of the MMF to adjust the specklegram size while maintaining the spatial correlation between the mechanical perturbations of MMF and the specklegram. The specklegram diameter after FOC was approximately 2 mm.

(3) Detection System: At the output end of the MMF, an imaging system consisting of a high-resolution CCD camera (pixel size of 1.85 µm, resolution of 4032 × 3037, and a photosensitive area of 7.4 mm × 5.6 mm) was used to capture the specklegrams. An attenuation was used to reduce the power of the laser, in order to capture clear specklegram patterns. The CCD camera was a laser beam quality analyzer produced by Wavelab Technology (Wavelab Technology Co., Ltd., Nanjing, China).

(4) Translation stage: A high-precision motorized stepper stage produced by Winner Optics (Winner Optics Technology Co., Ltd., Beijing, China) was used to generate the vertical displacement, with each step controlled via programming. The stage has a maximum travel range of 20 mm, with a max repeatable error of 2 µm. Additionally, the translation stage is equipped with an optical calibration system which provides high-precision displacement values. These values can be assessed in order to determine whether any displacement errors have occurred.

**Figure 1:**
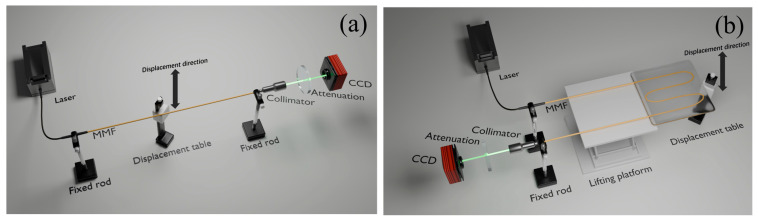
Experimental setup diagram. (**a**) Horizon fiber displacement. (**b**) S-shape displacement sensor.

Two experiments were designed to validate the performance of the sensor. In the first experiment, the optical fiber was positioned horizontally, and vertical displacement was applied at the midpoint using a translation stage. In the second experiment, the optical fiber was fixed into an S-shape on a plane and encapsulated in flexible material, and vertical displacement was applied at one end using a translation stage. The experimental setups are illustrated in Figure 1.

**Figure 2:**
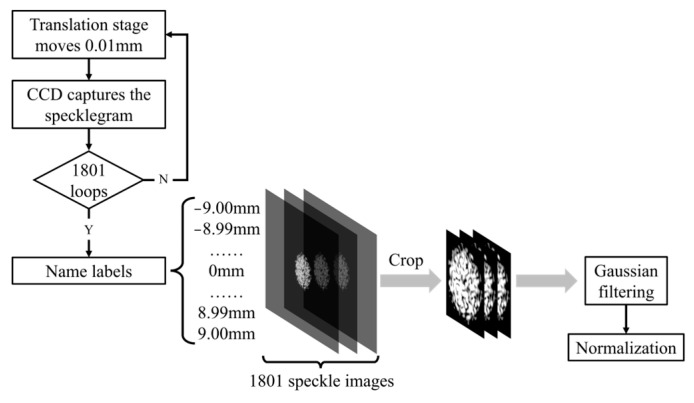
Process of acquiring the one-shot MMF specklegram dataset.

The captured images undergo several preprocessing steps to enhance their quality. Normalization is applied to adjust for lighting variations and improve contrast, while Gaussian filtering is employed to suppress artifacts. These artifacts primarily include high-frequency noise in the specklegram images, such as sensor readout noise from the CCD camera, environmental interference (e.g., stray light fluctuations), and minor laser instability. The Gaussian filter mitigates these artifacts by smoothing localized pixel intensity variations through weighted averaging, thereby preserving the structural integrity of speckle patterns and enhancing the signal-to-noise ratio for robust feature extraction during model training. The images are then cropped to focus on the region where the specklegram is the most prominent and stable. These preprocessed images are stored in a structured database, with each image linked to its corresponding displacement value. Unlike other methods referenced, each displacement corresponds to a unique specklegram image. The process of acquiring the specklegram datasets is illustrated in Figure 2.

### 2.2. Model Architecture

In this study, the ResNet-18 architecture is utilized; this is a variant of the deep ResNet introduced by He et al. [10]. The key innovation in ResNet is the introduction of residual learning, which enables the network to learn residual functions relative to the layer inputs, rather than learning unreferenced functions directly. Mathematically, the residual block can be expressed as

(2) $$H\left(x\right)=F\left(x,\left\{W_{i}\right\}\right)+x$$

where H(x) is the output of the residual block, $F\left(x,\left\{W_{i}\right\}\right)$ represents the residual mapping, and x is the input to the block. The term $\left\{W_{i}\right\}$ denotes the weights of the layers within the residual block. The identity mapping *x*, also referred to as the shortcut connection, is added directly to the output of the residual function $F\left(x,\left\{W_{i}\right\}\right)$, ensuring that the gradient can flow through the network more easily.

To further enhance the stability of the MMF specklegrams one-shot dataset training process, a residual scaling mechanism is introduced within each residual block. Residual scaling involves multiplying the output of the residual function $F\left(x,\left\{W_{i}\right\}\right)$ by a learnable residual scalar parameter *α* before adding it to the shortcut connection:

(3) $$H\left(x\right)=\alpha \cdot F\left(x,\left\{W_{i}\right\}\right)+x$$

The range of *α* is constrained between 0 and 1 to prevent the residual contribution from overwhelming the shortcut connection early in training. This scaling factor is trained alongside the network weights, allowing the model to dynamically adjust the contribution of the residual function throughout the training process. The learnable residual scaling improves the stability of the training process by allowing the network to make more nuanced adjustments to the residual contributions.

**Figure 3:**
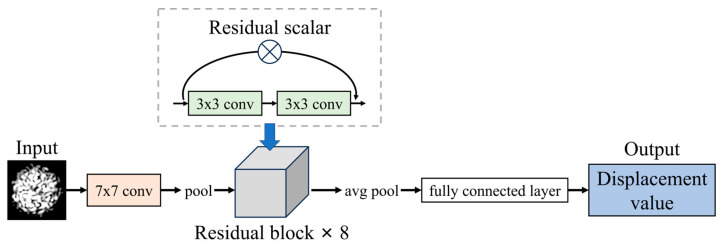
Structure of proposed model.

To adapt the model for a regression task, the final fully connected layer was modified to output a single continuous value rather than class probabilities. This modification enables the model to predict scalar values directly, which is essential for tasks such as displacement prediction. The structure of proposed model is shown in Figure 3.

The input to the network consists of grayscale images, which were converted to three channels by replicating the grayscale data across the RGB channels. This conversion ensures compatibility with the ResNet architecture, which expects three-channel input images. The images were resized to 126 × 126 pixels.

Mean Squared Error (MSE) was employed as the loss function, which is appropriate for regression tasks. The model was optimized using the Adam optimizer with a learning rate of 0.0001 to ensure stable convergence during training. The network was trained for 200 epochs with a batch size of 32. To ensure reproducibility of the results, a fixed random seed was used across all experiments. The dataset was split into training and validation sets, with 80% of the data used for training and 20% for validation.

To evaluate the trained model, several metrics were used, including Mean Absolute Error (MAE), Root Mean Squared Error (RMSE), Max Error ($\varepsilon_{max}$), and the R^2^ score, as defined below:

(4) $$MAE=\frac{1}{n}\sum_{i=1}^{n}\left|y_{i}-{\hat{y}}_{i}\right|$$

(5) $$RMSE=\sqrt{\frac{1}{n}\sum_{i=1}^{n}{\left(y_{i}-{\hat{y}}_{i}\right)}^{2}}$$

(6) $$\varepsilon_{max}=max\left(y_{i}-{\hat{y}}_{i}\right)$$

(7) $$R^{2}=1-\frac{\sum_{i=1}^{n}{\left(y_{i}-{\hat{y}}_{i}\right)}^{2}}{\sum_{i=1}^{n}{\left(y_{i}-{\bar{y}}_{i}\right)}^{2}}$$

where $y_{i}$ and ${\hat{y}}_{i}$ represent the predicted value and the applied displacement of the *i*-th specklegram, respectively. The n represents the number of specklegrams.

## 3. Results and Analysis

### 3.1. Experimental Results

**Figure 4:**
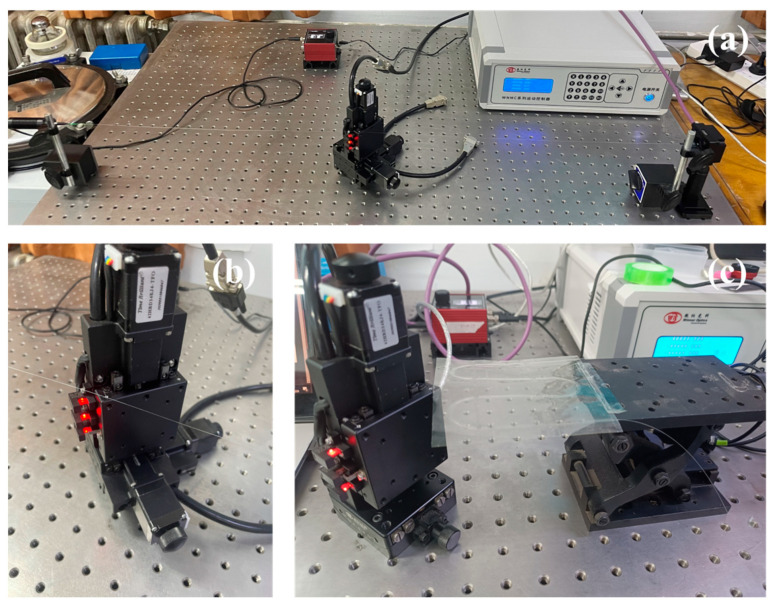
Displacement experiment: (**a**) horizontal fiber with midpoint vertical displacement; (**b**) translation stage mechanism; and (**c**) S-shaped flexible sensor with end displacement.

Two displacement-sensing experiments were conducted, as illustrated in Figure 4. In Figure 4a, the optical fiber is positioned in a horizontal stretching configuration, with a translation stage (shown in Figure 4b) placed at the midpoint of the fiber to induce vertical displacements ranging from −9 mm to 9 mm. In Figure 4c, the optical fiber is encapsulated in an S-shape within plastic tape, forming a planar flexible sensor. The translation stage is attached to one end of the flexible sensor, generating vertical displacements which also range from −9 mm to 9 mm.

In the horizontal optical fiber experiment, the joints of fiber at both ends were fixed at the same height. Additionally, both joints were slightly tensioned to ensure the fiber remained as horizontal as possible. A fiber-optic collimator was installed at the output end of the MMF to obtain a specklegram of the appropriate size. An attenuator was mounted on the CCD to ensure a clear specklegram, with sharp specklegrams at the edges and distinguishable contours in the high-intensity central region.

In both experiments, the initial position of the translation stage was −9 mm, and a displacement step size of 0.01 mm was used. A total of 1801 specklegrams were collected for each experiment. Although the initial positions of both the optical fiber and the S-shaped flexible sensor were horizontal, the final vertical displacement reached 18 mm. However, since the objective of the experiment was to measure the displacement of the stage, the vertical displacement of the stage, ranging from −9 mm to 9 mm, was used as the reference. The dataset was subsequently pre-processed as outlined in Figure 2.

### 3.2. Results

Table 1 compares the performance of the different initial values of the residual scaling factor α relative to model performance in the horizontal fiber displacement experiment, under both fixed and learnable settings. When α = 1, the model does not employ residual scaling. It can be observed that residual scaling improves the performance of the ResNet model. Through repeated testing, it was found that the optimal initial values of α were 0.75 for the horizontal optical fiber displacement experiment and 0.29 for the S-shaped flexible sensor displacement experiment, resulting in strong model performance in both cases.

**Figure 5:**
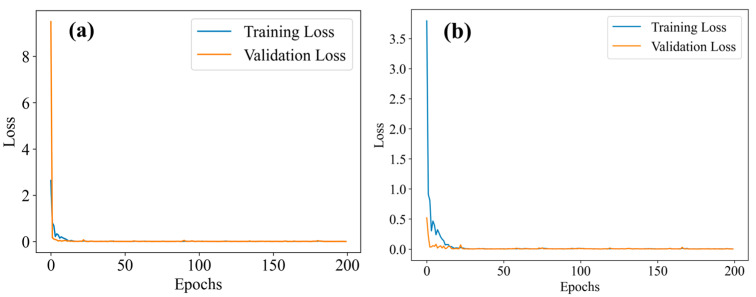
Loss curve of ResNet-18 based on residual scaling: (**a**) Horizon fiber (α = 0.75); (**b**) S-shape flexible sensor (α = 0.29).

**Figure 6:**
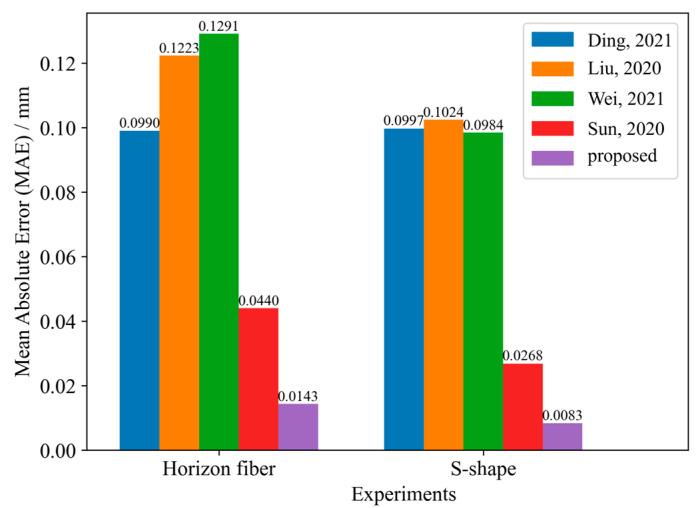
Correlation between predicted values and the applied displacement in the two experiments [7,9,13,17].

To demonstrate the effectiveness of the proposed method, the model was compared with other displacement sensors based on MMF, using two experimental datasets. The loss curves both reach the convergence state during the training process, as shown in Figure 5. To evaluate the predictive performance of the proposed method, a comparison was made with the models presented in the relevant literature on displacement sensors based on MMF. Based on the other methods described, we constructed the corresponding neural network model and input the collected speckle dataset to obtain the corresponding results. The comparison of the MAE of various models is shown in Figure 6. For the two experiments, the proposed method has a lower MAE, and thus a higher prediction accuracy than other deep learning methods. The regression evaluation metrics comparisons are summarized in Table 2 and Table 3.

The experimental results clearly demonstrate that the proposed model significantly outperforms the presently existing model. The residual scaling enables the model to focus on the most relevant features within the specklegrams, resulting in higher accuracy in displacement measurements. Additionally, the experimental results indicate that the S-shaped flexible sensor exhibits lower error, likely due to its more sensitive response to deformation, which improves sensitivity and accuracy.

**Figure 7:**
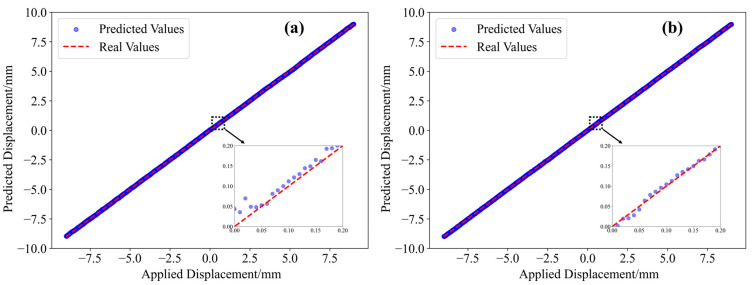
Displacement prediction results: (**a**) Horizon fiber (α = 0.75); (**b**) S-shape flexible sensor (α = 0.29).

The predictions of the 1801 displacement values in the two experiments are shown in Figure 7. It can be observed that the predicted values closely match the actual displacement values.

**Figure 8:**
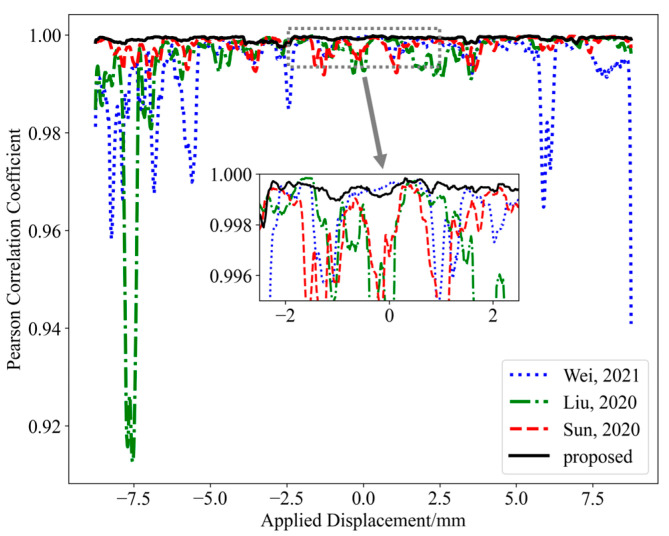
Correlation between predicted values and applied displacements for the S-shaped flexible sensor [7,9,13].

Additionally, the relationship between the predicted and applied displacement is investigated by calculating correlation coefficients using a sliding window. First, the Pearson correlation coefficient for each segment of data is computed, where the window size determines the number of data points considered in each calculation (a window size of 50 is used in this paper). The Pearson correlation coefficient r can be calculated as the ratio of the covariance of the predicted and actual values to the product of their standard deviations, thereby quantifying the linear relationship between the two variables, as defined in Equation (8). The correlation between predicted values and applied displacements of the S-shaped flexible sensor was compared, as shown in Figure 8. The results indicate that the predicted displacement values obtained by the proposed method exhibit a better correlation with the applied displacement, demonstrating the effectiveness of the method in the one-shot MMF specklegram prediction task.

(8) $$r=\frac{\sum \left(x_{i}-\bar{x})(y_{i}-\bar{y}\right)}{\sqrt{\sum {\left(x_{i}-\bar{x}\right)}^{2}\sum {\left(y_{i}-\bar{y}\right)}^{2}}}$$

### 3.3. Analysis of Residual Scaling

**Figure 9:**
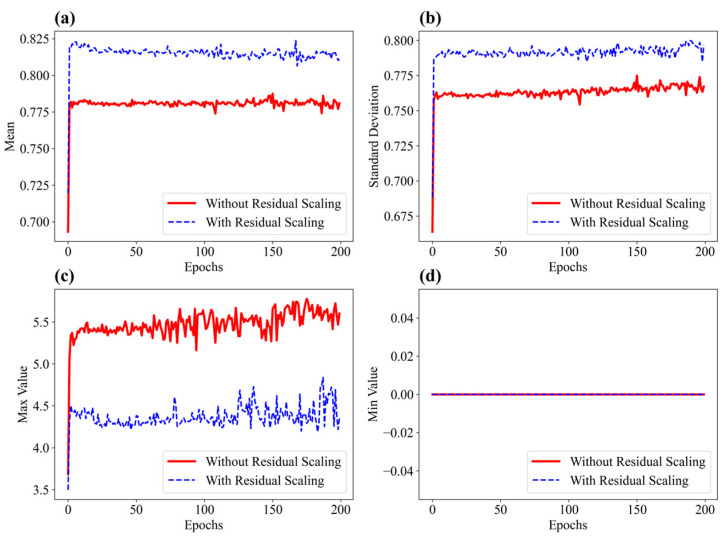
Statistics for the feature maps extracted from layer 1 over epochs: (**a**) mean of feature maps; (**b**) standard deviation of feature maps; (**c**) max value of feature maps; and (**d**) min value of feature maps.

At the end of each epoch, feature maps were extracted from layer 1 of the S-shaped flexible sensor, both with residual scaling (α = 0.29) and without residual scaling, and their statistics (mean, standard deviation, maximum, and minimum) were calculated. The variation curves of these statistics were plotted for comparison, as shown in Figure 9.

It can be observed that the model with residual scaling exhibits a slightly higher mean in the feature maps during training, and higher mean activation intensities typically indicate that neurons in the layer are more responsive to inputs [24]. This suggests that the model’s activation intensity is generally higher, reflecting a stronger response to the specklegram images during feature extraction. In deep neural networks, a higher mean often indicates that the model is more actively engaging with certain features at specific layers. This heightened response implies greater sensitivity in capturing critical information from the MMF specklegrams, such as edges, intensity, or more complex high-level features. A moderate increase in the mean suggests that the model is responding more comprehensively to the specklegrams, covering a broader range of information rather than excessively activating specific features. Over-activation could lead to the model favoring certain features while neglecting others. However, a balanced, moderate increase in the mean helps ensure that the model captures diverse features without overemphasizing any particular aspect of the input.

The model with residual scaling also exhibits a higher standard deviation in the feature maps, indicating greater diversity in activation values and suggesting that the model maintains a higher degree of variability in feature extraction. A higher standard deviation generally indicates that the model is capable of recognizing more details and variations of specklegrams during feature extraction. This heightened response suggests improved sensitivity to subtle specklegram variations induced by sub-millimeter displacements. The increased standard deviation with residual scaling further corroborates this interpretation, as greater activation diversity reflects enhanced discrimination of fine-grained speckle features [25].

Additionally, the model with residual scaling exhibits lower maximum values in the feature maps, suggesting more controlled activation levels. This helps prevent excessively high activations and reduces the risk of gradient explosion. From the minimum value plots, it can be observed that, regardless of whether residual scaling is employed, the minimum value of the feature maps consistently remains at zero. This consistency indicates that the lower bound of activation values in this layer does not undergo significant variations during training.

These observations are consistent with a mechanism of residual scaling: by adaptively weighting residual contributions, the network emphasizes informative feature transformations while preserving stable gradient flow through shortcut connections [26].

### 3.4. Generalization Validation

**Figure 10:**
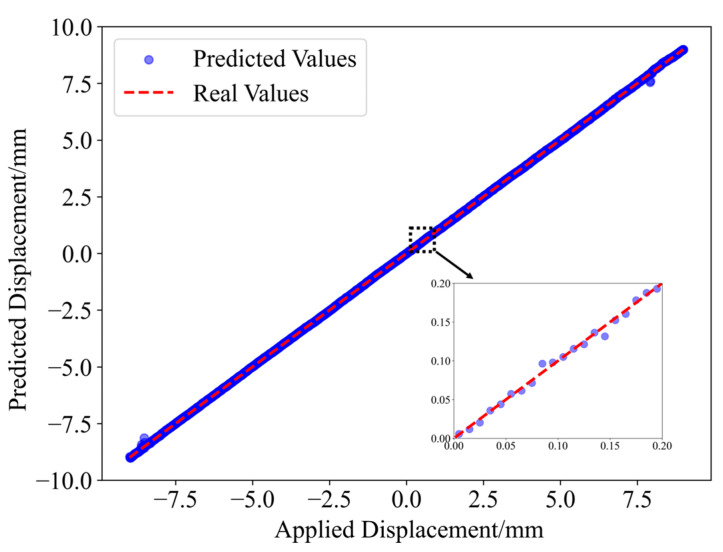
Prediction results for the 0.005 mm displacement resolution (α = 0.27).

Furthermore, the model was subjected to generalization-based validation. With vertical displacement applied to an S-shaped flexible sensor, a one-shot MMF specklegram dataset was collected with a displacement step size of 5 μm, obtaining 3601 specklegram images over a displacement range of 18 mm. The specklegrams corresponding to displacements of −9.00 mm, −8.99 mm, −8.98 mm, …, 8.98 mm, and 9.00 mm were selected and divided into a training set and a test set with an 8:2 split. Specklegrams corresponding to displacements of −8.995 mm, −8.985 mm, −8.975 mm, …, 8.985 mm, and 8.995 mm were selected for the generalization-based validation. The residual scaling factor α was set to 0.27. The trained model was used to make predictions on these specklegrams. The error analysis associated with these predictions is presented in Table 4, and the comparison between the predicted and actual values is shown in Figure 10. As can be observed, the model proposed in this study further improves displacement prediction accuracy, improving upon other models.

## 4. Temperature Noise and Immunity

**Figure 11:**
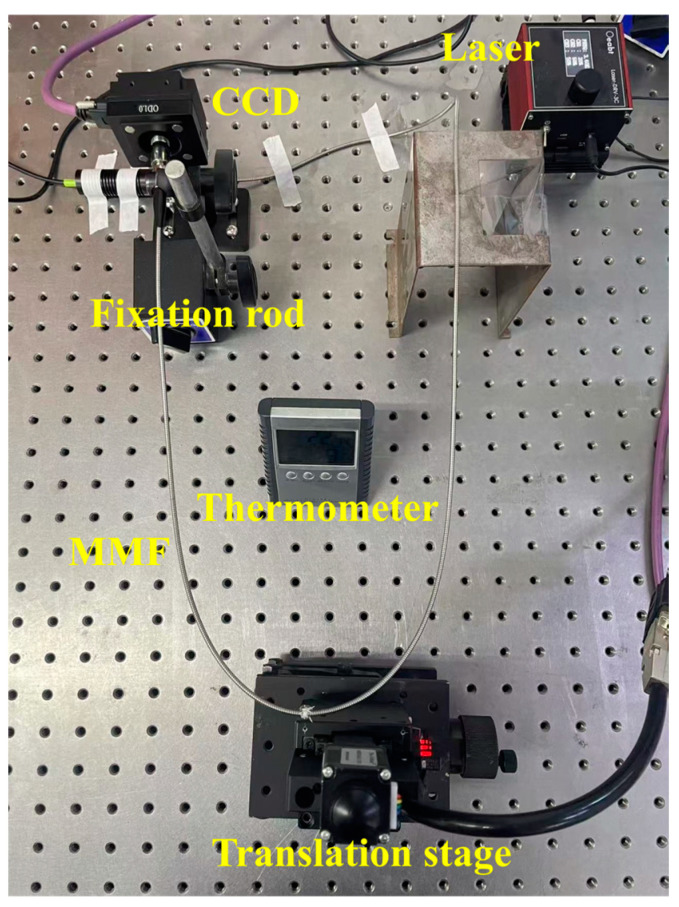
Schematic diagram of the experimental setup for MMF sensing stability and repeatability analysis.

Further experiments were conducted on the stability of MMF sensing under temperature variations. Temperature-induced specklegram distortions have been reported in both static [27] and dynamic [28] sensing scenarios. It is crucial to eliminate the nonlinear effects caused by environmental interference [29]. To minimize interference caused by external factors such as vibrations, armored MMF fibers were used for enhanced mechanical stability, whereas bare fibers were adopted in previous experiments under controlled environments. The armored structure effectively isolates external vibrations from human activities or accidental collisions, ensuring that strain variations originate solely from displacements and ambient temperature fluctuations. In this setup, the MMF was only affected by strains induced by displacement and environmental temperature. The experimental setup is shown in Figure 11. The MMF is suspended in a U-shape on a translation stage, and reciprocating vertical displacement is applied at the midpoint of the bend with a step size of 0.01 mm, from −9 mm to 9 mm, and then from 9 mm to −9 mm. A total of 3602 specklegrams were collected for the reciprocating displacement of one group. At the same time, in order to reduce the temperature differences during the collection process for each group, the translation stage moves continuously at 0.01 mm/s, and the CCD continuously captured a specklegram every one second. This method can also be used to analyze the displacement-sensing capability of MMF under dynamic displacement. The thermometer was placed at the center of the experimental apparatus to measure the ambient temperature.

**Figure 12:**
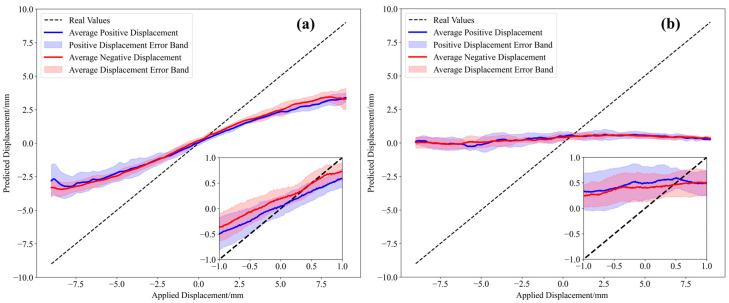
Displacement predictions under different temperatures: (**a**) room temperature of 22.3 °C; (**b**) room temperature of 21.3 °C.

Under an average room temperature of 22.2 °C, five groups of specklegrams were captured for model training. Additionally, five groups of specklegrams were captured at average room temperatures of 22.3 °C and 21.3 °C, respectively. The model was trained at 22.2 °C, and was then used to perform displacement prediction on the other two groups of specklegram data. The results are shown in Figure 12. As shown in Figure 12a, with a minor temperature variation of 0.1 °C, the average fitting slope is 0.43158, indicating a significant prediction error, yet the data maintains robust linearity (R^2^ = 0.9778) and a dispersion of 5.26%. As shown in Figure 12b, the sensor exhibits no response to displacement variations when subjected to a temperature change of approximately 1 °C. The results indicate that the MMF displacement sensor exhibits high noise-to-temperature variations; therefore subsequent research efforts should focus on enhancing its temperature immunity to improve its generalization capability and environmental adaptability.

**Figure 13:**
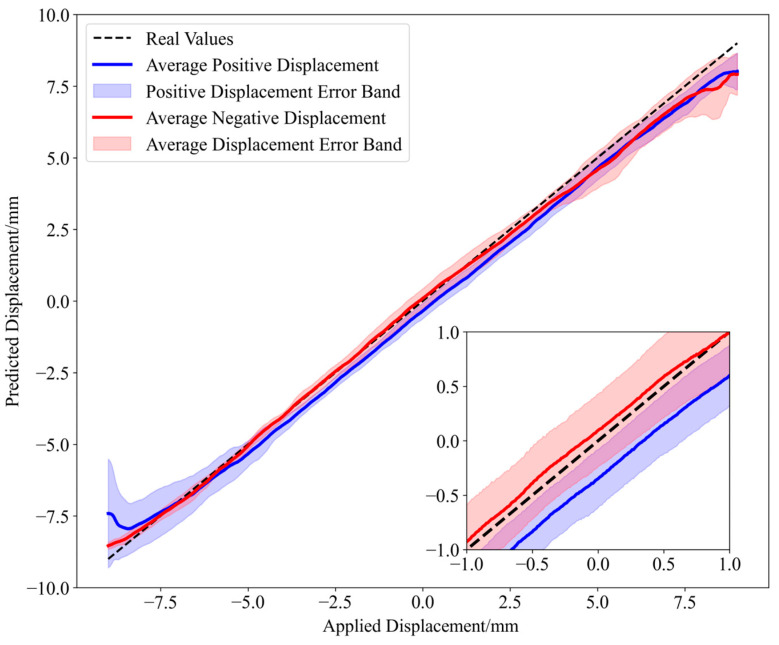
Displacement predictions under temperatures of 21.3 °C to 22.35 °C.

A total of 20 groups of specklegrams were captured under the different temperatures. The temperatures of each group are shown in Table 5. To minimize the influence of temperature, several specklegram datasets were selected for model training based on temperature-ordered intervals (groups: 1, 3, 5, 7, 9, 11, 13, 15, 17, and 19), with the residual scaling factor set to 0.5. This approach enables the model to learn MMF speckle patterns under varying temperature conditions. Meanwhile, the generalization ability and the sensor’s repeatability under the different temperature environments were tested. The results are shown in Figure 13.

The repeatability analysis of the sensor was conducted by calculating the dispersion *D*, with the calculation formula as follows:

(9) $$s^{2}=\frac{1}{n-1}\sum_{i-1}^{n}{\left(y_{i}-{\bar{y}}_{i}\right)}^{2}$$

(10) $$D=\frac{2s}{R}\times 100\%$$

where $y_{i}$ represents the predicted value of the *i*-th specklegram, ${\bar{y}}_{i}$ is the predicted mean value of *i*-th specklegram in all groups, *s* is the sample variance, and *R* is the measurement range of the displacement sensor. With a dispersion of 5.08% and within a measurement range of 18 mm, this indicates that the data within the ±2 standard deviation range account for 5.08% of the measurement range. This suggests that the displacement sensor based on the MMF specklegram is relatively concentrated, and multiple sampling demonstrates good consistency.

Based on the statistical formulas for evaluating sensor performance, the nonlinearity was calculated as the ratio of maximum prediction error to measurement range, and hysteresis as the maximum path deviation. 

Specifically, the nonlinearity of each path is defined as

(11) $$\delta_{L}=\frac{\max\left(\left|{\bar{y}}_{i}-{\hat{y}}_{i}\right|\right)}{R}\times 100\%$$

The hysteresis is calculated as

(12) $$\delta_{H}=\frac{\max\left(\left|{\bar{y}}_{positive}-{\bar{y}}_{negative}\right|\right)}{R}\times 100\%$$

where ${\bar{y}}_{positive}$ represents the predicted mean value of positive displacement in all groups, and ${\bar{y}}_{negative}$ represents the predicted mean value of negative displacement in all groups.

Based on Equations (11) and (12), the nonlinearity of the positive path was determined to be 8.71%, while the nonlinearity of the negative path was 6.71%. Additionally, the hysteresis was measured at 6.13%. According to Figure 13 and the statistical results associated with the performance of the sensor, although environmental temperature interference still exists, the proposed method can be effectively applied to high-resolution and wide-range MMF displacement sensor through one-shot sampling under the different temperature conditions.

The error evaluation for each model is shown in Table 6. As observed in this table, the proposed model in this study exhibits the lowest errors under varying environmental conditions. This demonstrates the superiority of the residual scaling method in speckle pattern prediction based on MMF.

## 5. Conclusions

This paper proposes a high-resolution and wide-range displacement-sensing method based on MMF specklegrams and deep learning. By constructing a one-shot dataset with 1801 displacement values and a resolution of 0.01 mm and employing a ResNet-18 model with residual scaling, the study significantly improves the accuracy and stability of displacement prediction.

The main contributions of this work are as follows: (1) The use of a one-shot dataset simplifies the data collection process and improves the resolution and measurement range of the MMF-based displacement sensor. (2) The residual scaling applied in ResNet-18 significantly enhances the accuracy of displacement prediction. (3) The experimental results show that the proposed method not only maintains high accuracy over a wide displacement range but also performs well in higher-resolution predictions. (4) The displacement-sensing measurement of MMF under temperature variations was achieved.

The experimental results demonstrate that the proposed model outperforms traditional MMF specklegram-based displacement-sensing methods in both accuracy and measurement range. Specifically, the proposed method achieves an average prediction error as low as 0.0083 mm, with a measurement range of 18 mm, which is significantly better than other CNN-based methods. Moreover, the model shows a strong generalization ability when tested on a 0.005 mm resolution dataset, further validating its potential for high-precision displacement prediction. Several groups of specklegrams were captured through one-shot sampling under the different temperatures, and the trained model effectively predicted displacements under varying temperature conditions. The model achieved displacement sensing over an 18 mm measurement range with a dispersion of 5.08%, an average nonlinearity of 7.71% and a hysteresis of 6.13% under the temperature ranging from 21.25 °C to 22.35 °C.

In summary, this research demonstrates the potential of one-shot MMF specklegram prediction with deep learning techniques, particularly through the use of residual scaling, to significantly enhance displacement-sensing performance. The findings offer a solid foundation for advancing MMF-sensing technologies, with potential applications in areas such as structural health monitoring and precision metrology. Future research could focus on further improving the precision of the translation stage to achieve even higher resolution sensing, and on enhancing model robustness through one-shot specklegram sampling in varied environments to enable practical applications.

## Figures and Tables

**Table 1:** MAE of model under different residual scaling factors.

Horizon Fiber		S-Shaped Flexible Sensor	
α	MAE/mm	α	MAE/mm
0.1	0.0203	0.1	0.0228
0.2	0.0325	0.2	0.0168
0.3	0.0279	0.3	0.0127
0.4	0.0255	0.4	0.0240
0.5	0.0260	0.5	0.0459
0.6	0.0354	0.6	0.0483
0.7	0.0239	0.7	0.0274
0.8	0.0157	0.8	0.0220
0.9	0.0408	0.9	0.0237
1	0.0435	1	0.0268

**Table 2:** Displacement sensors: comparative analysis (Horizon fiber).

Reference	Backbone	MAE/mm	RMSE/mm	Max Error/mm	R^2^
Liu [7]	VGG	0.0990	0.1262	0.4956	0.999425
Li [11]	VGG	0.1223	0.1623	0.4682	0.999048
Wei [13]	CNN	0.1291	0.1669	0.4850	0.998994
Sun [9]	ResNet18	0.0440	0.0548	0.1612	0.999892
Proposed	ResNet18	0.0143	0.0185	0.0612	0.999988

**Table 3:** Displacement sensors: comparative analysis (S-shape).

Reference	Backbone	MAE/mm	RMSE/mm	Max Error/mm	R^2^
Liu [7]	VGG	0.0997	0.1322	0.5465	0.999465
Li [11]	VGG	0.1024	0.1177	0.2588	0.999500
Wei [13]	CNN	0.0984	0.1316	0.4696	0.999374
Sun [9]	ResNet18	0.0268	0.0375	0.1229	0.999949
Proposed	ResNet18	0.0083	0.0109	0.0397	0.999996

**Table 4:** Prediction error for the 0.005 mm displacement resolution.

Reference	Backbone	MAE/mm	RMSE/mm	Max Error/mm	R^2^
Liu [7]	VGG	0.2453	0.3115	1.5853	0.996496
Li [11]	VGG	0.0753	0.0936	0.2712	0.998710
Wei [13]	CNN	0.0754	0.1027	0.4651	0.999616
Sun [9]	ResNet18	0.0266	0.0415	0.4056	0.999938
Proposed	ResNet18	0.0138	0.0322	0.3960	0.999963

**Table 5:** The dataset for the 20 groups of specklegrams under different temperatures.

Group	Temperature
1	21.2 °C
2	21.3 °C
3	21.3 °C
4	21.3 °C
5	21.4 °C
6	21.4 °C
7	21.5 °C
8	21.5 °C
9	21.5 °C
10	21.65 °C
11	21.75 °C
12	22.0 °C
13	22.1 °C
14	22.2 °C
15	22.2 °C
16	22.2 °C
17	22.2 °C
18	22.25 °C
19	22.3 °C
20	22.35 °C

**Table 6:** Performance of models under the different temperatures.

Reference	Backbone	MAE/mm	RMSE/mm	Max Error/mm	R^2^
Liu [7]	VGG	4.4496	5.1773	10.6697	0.0083
Li [11]	VGG	4.7751	5.6494	11.3050	−0.1808
Wei [13]	CNN	0.5108	0.7384	6.9802	0.9721
Sun [9]	ResNet18	0.4204	0.6236	5.9725	0.9852
Proposed	ResNet18	0.3952	0.5792	5.5952	0.9876

## Data Availability

The data that support the findings of this study are available on request from the corresponding author.
